# Bioinspired Self-assembling Peptide Hydrogel with Proteoglycan-assisted Growth Factor Delivery for Therapeutic Angiogenesis

**DOI:** 10.7150/thno.35803

**Published:** 2019-09-21

**Authors:** Lu-Chieh Huang, Huan-Chih Wang, Liang-Hsin Chen, Chia-Yu Ho, Pei-Hsuan Hsieh, Ming-Yuan Huang, Hsi-Chin Wu, Tzu-Wei Wang

**Affiliations:** 1 Department of Materials Science and Engineering, National Tsing Hua University, Hsinchu, Taiwan; 2 Division of Neurosurgery, Department of Surgery, National Taiwan University Hospital, Taipei, Taiwan; 3 College of Biological Science and Technology, National Chiao Tung University, Hsinchu, Taiwan; 4 Department of Medicine, Mackay Medical College, New Taipei City, Taiwan; 5 Department of Emergency, Mackay Memorial Hospital, Taipei, Taiwan; 6 Department of Materials Engineering, Tatung University, Taipei, Taiwan

**Keywords:** self-assembling peptide, proteoglycan, growth factors, intrinsic crosslinking, angiogenesis

## Abstract

Critical challenges still exist in surgical theaters and emergency rooms to stop bleeding effectively and facilitate wound healing efficiently. In circumstances of tissue ischemia, it is essential to induce proper angiogenesis to provide adequate vascular supply to the injury site.

**Methods**: In view of these clinical unmet needs, we propose an applicable approach by designing functionalized self-assembling peptide (SAP) hydrogel with two sequences of RADA16-GGQQLK (QLK) and RADA16-GGLRKKLGKA (LRK) in this study. The SAP hydrogel conjugated with QLK functional motif could be crosslinked by endogenous transglutaminase, one of the intrinsic factors secreted during the coagulation process, the mechanical property of the hydrogel can then be enhanced without the need of external support. On the other hand, the LRK sequence exhibited a good binding affinity with the proteoglycan heparan sulfate and could act as a cofactor by sustaining the release of embedded growth factors.

**Results:** The results showed that this SAP solution underwent self-assembling process in a physiological environment, formed hydrogel * in situ*, and possessed good shear thinning property with injectability. After pH adjustment, the SAP developed densely-compacted fiber entanglement that closely mimicked the three-dimensional fibrous framework of natural extracellular matrix. Such scaffold could not only support the survival of encapsulating cells but also promote the capillary-like tubular structure formation by dual angiogenic growth factors. The *ex ovo* chicken chorioallantoic membrane assay demonstrated that the growth factor-loaded hydrogel promoted the sprout of surrounding vessels in a spoke-wheel pattern compared to growth factor-free counterparts.

**Conclusion:** The designer bioinspired SAP hydrogel may be an attractive and promising therapeutic modality for minimally-invasive surgery, ischemic tissue disorders and chronic wound healing.

## Introduction

Effective hemostasis is crucial for invasive surgical or interventional procedures, especially in major trauma or when dealing with hypervascular lesions. Ineffective hemostasis leads to an increased amount of intra- or post-operative blood loss and further results in undesired morbidity or mortality 1. Although multiple therapeutic hemostatic adjuncts exist, finding a more efficient strategy to arrest active bleeding is still a challenge 2. After hemostasis, the wound healing process will move to the next stage, including inflammation, proliferation and remodeling phases. In the proliferation phase, angiogenesis plays a central role in supplying growth factors (GFs), cytokines, chemokines, and circulating progenitor cells that are essential to recapitulate normal tissue functions 3. It is especially important to induce adequate angiogenesis in conditions of ischemic diseases and chronic non-healing wounds such as diabetic foot ulcers, pressure sores, and peripheral vascular diseases 4. GFs are soluble secreted proteins that have the ability to regulate cellular behaviors and to guide tissue renewal 5; among all GFs, only recombinant platelet-derived growth factor (PDGF) has currently been approved by the FDA for enhancing the healing of diabetic ulcers 6. Unfortunately, the required high dosage of PDGF increases the risks of neoplastic growth and systemic side effects. Other limitations of PDGF bolus injection include the low efficiency for drug delivery and the poor drug stability against proteases. In view of these problems, the purpose of this study is to develop a hydrogel-based modality that serves as a more effective and safer hemostatic agent. The agent will also have the abilities to control the release of encapsulated therapeutic factors in an extended timeframe, to trigger neovascularization, and to restore normal angiogenesis in our body.

Preserving the active functions of GFs and applying non-covalent binding interactions to sequester GFs are two important issues to address. Heparan sulfate (HS), one of the major subgroups of glycosaminoglycans (GAGs) in the extracellular matrix (ECM), binds GFs through conformational configuration and electrostatic attraction force 7. This association plays an important role in stabilizing the bioactivity of GFs, protecting GFs from enzymatic clearance, and modulating GF receptor signaling pathways 8.

Self-assembly is an inherent phenomenon commonly observed in natural biological systems. In recent years, several rationally designed peptide sequences have been developed through the concept of hierarchical self-assembly 9. The scientific advantages of well-designed peptides include not only the ECM-like biomimetic three-dimensional architecture but that their degradation products are amino acids which prevented the hazards of animal-derived pathogens. Moreover, the *in situ* sol-gel transition occurs at physiological conditions so that the jelly-like hydrogel could be easily conformed to lesion cavities 10, 11. Despite many attracting features mentioned above, the major limitation of self-assembling peptide (SAP) materials is the relatively low mechanical properties due to the weak non-covalent bond interactions 12. In other words, the fragile scaffolds hinder the applications of SAP hydrogel. Therefore, how to enhance the mechanical properties of the peptides and to tether therapeutic proteins to the peptide sequences are critical issues for the development of advanced SAP materials. In view of this, many different cross-linking approaches have been proposed 13-16. However, most of the studies have been documented with concerns of cytotoxicity from chemical cross-linkers or UV irradiation. Chemical crosslinking is a fast and versatile approach to create hydrogel scaffold, but the chemical reactions and the toxic cross-linkers often pose potential harm for cell survival in the formed gel. When applying UV-polymerization strategy, the dysfunction of encapsulated proteins and drugs is a serious problem.

**Figure 1:**
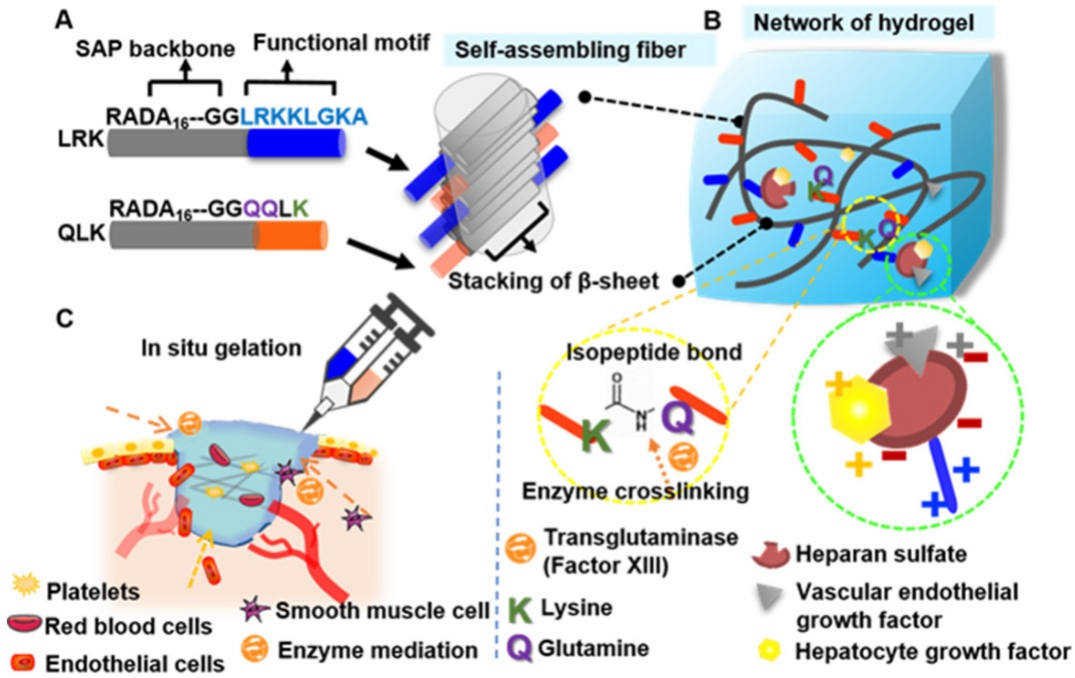
** The proposed self-assembling mechanism for functionalized peptide sequences**. (A-B) After direct conjugation of functional motifs to RADA_16_ backbone, the protruded functional motifs will be part of the fibrous network which mimic the biomechanical and biochemical properties of extracellular matrix at the same time. (C) The schematic illustration of injectability and * in situ* gelation properties of designer SAP; moreover, it can not only achieve hemostasis immediately but also restore optimal angiogenesis of damaged sites.

In this study, we propose an applicable approach by designing a functionalized self-assembling peptide (fSAP) hydrogel with two tailor-designed sequences, RADA_16_-GGQQLK (QLK) and RADA_16_-GGLRKKLGKA (LRK), respectively. Two glycines (G) are added on the C-terminus as a spacer to guarantee the flexibility and a correct exposure of the conjugated motifs to receptors (Figure 1A). After the mediation of transglutaminase, *i.e.* factor XIIIa in the coagulation cascade, the side chains of glutamine (Q) and lysine (K) on RADA_16_-GGQQLK can be crosslinked through acyl transfer reaction to the further formation of intermolecular ε-(γ-glutamyl) lysine isopeptide bonding. This covalent crosslinking process is able to resist proteolytic degradation and is a relatively fast crosslinking reaction 17. Herein, the improvement in mechanical stability and the enhancement of storage modulus of hydrogel can be achieved. On the other hand, the other functional motif LRKKLGKA flanking around the self-assembling fibers has the ability to sequester the complexes of HS and angiogenic GFs, such as vascular endothelial growth factor (VEGF) and hepatocyte growth factor (HGF) 18. The cooperative binding process can act as a cofactor to facilitate angiogenesis synergistically (Figure 1B).

In summary, the fSAP solution can be co-delivered with transglutaminase by injecting directly to the wound to achieve effective hemostasis. Subsequently, sustained and localized release of the incorporated GFs, namely VEGF and HGF, synergistically enhance the therapeutic effect and govern the cell fate on ischemic tissue (Figure 1C). The novelty and significance of this study are: (1) the microstructure of self-assembling QLK/LRK peptide hydrogel not only mimics the natural ECM to recapitulate normal tissue integrity but also immobilizes essential GAGs and GFs through functional motifs; (2) the strength of hydrogel can be modulated by transglutaminase crosslinking process to prolong the degradation rate; (3) the spatiotemporal controlled release of VEGF and HGF can stimulate angiogenesis, enhance vascular perfusion and recapitulate normal tissue function of chronic non-healing wounds. We expect that the design and strategies demonstrated in this study provide a potential hydrogel-based treatment for conditions such as surgical hemostasis, chronic ulcer, critical limb ischemia, ischemic stroke, and peripheral vascular disease. Furthermore, fSAP hydrogel can also serve as a suitable reservoir for the delivery of injectable stem cells or therapeutic biological molecules (genes/drugs), making it an attractive biomedical material with a wide range of potential applications.

## Methods

### Materials

HS was purchased from YickVic. Sodium hydroxide (NaOH, Cat. no. MA-7708-69) was purchased from Mallinckrodt Pharmaceuticals. Hydrochloric acid (HCl, Cat. no. RD-30721) was purchased from Riedel-de Haën. Sucrose (Cat. no. JT-4072-01) and Glacial acetic acid (Cat. no. JT-9508-01) were purchase from JTBaker. Ninhydrin reagent solution (Cat. no. N 7285), endothelial cell growth supplement (ECGS, Cat. no. SI-E2759-15MG), heparin sodium (Cat. no. SI-H3393-100KU), Triton X-100 (Cat. no. SI-T8787), and Tween 20 (Cat. no. SI-P7949) were purchased from Sigma-Aldrich. Dulbecco's Modified Eagle Medium (Cat. no 11965092), bovine serum albumin (BSA, Cat. no. 30063481), Medium 199 (Cat. no. 11043023), penicillin-streptomycin (PS, Cat. no. 15140122), neural basal medium (Cat. no. 21103049), and LIVE/DEAD Viability/Cytotoxicity kit (Cat. no. L-3224) were purchased from Gibco. Matrigel was purchased from Corning (Cat. no. 354234). Fetal bovine serum (FBS, Cat. no. 04-001-1A) was purchased from Biological Industries. CytoTox 96® Non-Radioactive Cytotoxicity Assay was purchased from Promega (Cat. no. G1780). Recombinant Human VEGF165 (Cat. no. 100-20) and Recombinant Human HGF (HEK293 derived, Cat. no. 100-39H) were purchased from PeproTech. Human VEGF Quantikine ELISA Kit (Cat. no. DVE00) and Human HGF Quantikine ELISA Kit (Cat. no. DHG00) were purchased from R&D Systems. Hematoxylin Gills (Cat. no. SU01522) and Eosin (Cat. no. SU01602) were purchased from Leica.

### Preparation of fSAP

RADA_16_-QLK (AcN-RADARADARADARADAGGQQLK-NH_2_) and RADA_16_-LRK (AcN-RADARADARADARADAGGLRKKLGKA-CONH) with purity > 85 % were purchased from biopharmaceutical companies (Biosmart, Genscript) and used without further purification. The purity of the peptides was confirmed through high-performance liquid chromatography (HPLC). Identity of the peptides was also analyzed by mass spectrometry (MS). All aqueous peptide solutions were dissolved using Milli-Q water (18.2 MΩ) which was sterilized by an autoclave, stored at 4 °C and sonicated for at least 30 min before usage.

### Physiochemical Property Analyses of fSAP

#### Circular Dichroism Spectroscopy

Circular dichroism (CD) spectra were collected on a CD Spectrometer Model 410 (AVIV Biomedical Inc.) to explore the secondary structure of SAPs. The cuvette quartz cell's path length was 1mm with a wavelength range between 190-260 nm at ambient temperature. The 0.05 % (w/v) RADA_16_-QLK, RADA_16_-LRK, and HS solution were allowed to store at 4 °C overnight to ensure complete self-assembly before measurement.

#### Transmission Electron Microscopy and Atomic Force Microscopy

Transmission electron microscopy (TEM) was used to observe the microstructure of peptides with a Hitachi HT7700 (120 kV) Bio-TEM. The 0.05 % (w/v) RADA16-QLK before and after microbial transglutaminase (mTG) crosslinking were prepared at pH 7. The TEM samples were prepared by dropping 10 μL of peptide solution on the copper grid coated with carbon film and stained with 2 % phosphotungstic acid solution. The morphology of the peptide scaffolds was captured using atomic force microscopy (AFM) (DI Dimension 3100, Veeco Instruments Inc, US) in dynamic force mode. In brief, 0.05 % (w/v) peptide solution was sprayed on the surface of a cleaved mica for 5 min, the surface was then washed with sterilized water for several times and blown dry by anhydrous nitrogen.

#### Rheology

The mechanical properties of the hydrogel scaffold were measured using a rheometer (Anton Paar, US). Measurements for the dynamic oscillatory frequency sweep (0.1-100 Hz) were performed at 1% strain amplitude and 25 °C. Shear recovery (1 % strain for 60 s, 50 % strain for 30 s, 1 % strain for 60 s) was conducted for three cycles at 25 °C. All measurements were performed on a 25-mm cone plate and the Anton Paar Rheometer software was used for analysis.

### Purification and Bioactivity Quantification of mTG

mTG was purified from TG-K powder (Ajinomoto) by centrifuge purification using Amicon Ultra-15 centrifugal filter units (30 kDa). The working solution contained 0.5 M KH_2_PO_4_ (pH = 4.3, JTBaker), 0.5 M Na_2_HPO_4_.7H_2_O (pH = 8.7, Sigma-Aldrich), and 2 mM EDTA (Sigma-Aldrich). The purified solution was stored at -20 °C for further usage. The activity of purified mTG was confirmed by mTG assay kit (Zedira Z009).

### fSAP Hydrogel Formation, Crosslinking Process, and *in vitro* Disintegration Profile

The 2 % (w/v) RADA_16_-QLK and RADA_16_-LRK peptide solutions were mixed in a volume ratio of 3:1, and then introduced into a self-made cylinder mold. PBS buffer or cell culture medium was further added to trigger sol-gel transition. For the crosslinking process, the self-assembled cylindrical hydrogel was soaked into mTG solution (20 U/mL PBS) for 12 h at 38 °C. 40 µL of peptide hydrogel was transferred into a 96-well plate followed by 200 µL of PBS added to each well and incubated at 37 °C. At predetermined time points (3, 12, 24, 72, 168, 336, 504, 672, and 840 h), the supernatant was completely removed by syringe and replenished with fresh PBS. The collected supernatant was then stored at -80 ^o^C for further tests. Ninhydrin reagent solution was employed to quantify the disintegrated peptide amino acid sequences of the hydrogel according to manufacturer's protocols. After the addition of 95 % ethanol to stop the reaction, the absorbance of the hydrogel at 570 nm was measured by a SpectraMax Plus 384 Microplate Reader.

### Isothermal Titration Calorimetry

Isothermal titration calorimetry (ITC) measurements were operated at 25 °C using a Micro ITC-200 microcalorimeter (GE Healthcare Life Sciences). The procedure was performed by titrating 0.35 mM HS from a syringe into 0.2 mM RADA_16_-LRK peptide solution loaded in a cell (both in PBS). In the next step, the data were integrated after background correction and fitted to a curve to obtain the binding constant.

### Zeta Potential and Dynamic Light Scattering (DLS)

The zeta potential and hydrodynamic diameter of GF/HS complexes were recorded on a Malvern zeta-sizer (Malvern Instruments, UK). HS was complexed with VEGF or HGF, both at mass ratio of 100:1, to ensure that all available GFs could be bound by the solution saturated with HS.

### GF Encapsulating Efficiency and Release Profile

The release profiles of VEGF and HGF were investigated for 28 days. Each 100 µL of peptide hydrogel containing 50 ng of VEGF or HGF was immersed in the release buffer (PBS w/ 0.05 % Tween 20, 1 % BSA) at 37 °C. At specific time intervals, the supernatants were collected, stored at -80 °C, and fresh release buffer was compensated. VEGF ELISA kit and HGF ELISA kit (R&D Systems, US) were performed to measure the amount of released GFs in the supernatants following manufacturer's protocols. After adding the stop solution, the absorbance of the hydrogel at 450/540 nm was recorded by SpectraMax Plus 384 Microplate Reader.

### Culture of Cells in 2D and 3D Peptide Hydrogel

The human umbilical vein endothelial cells (HUVECs, H-UV001) and the embryonic fibroblast cells (NIH3T3) were purchased from the Bioresource Collection and Research Center (BCRC, Taiwan). HUVECs were carefully cultured in M199 medium supplemented with 10 % (v/v) FBS, 1 % (v/v) PS, 25 U/mL heparin, and 30 μg/mL ECGS. NIH3T3 cells were cultured in Dulbecco's Modified Eagle Medium (DMEM) with 10 % (v/v) FBS and 1 % (v/v) PS. Cells were cultured in the humidified incubator at 37 °C with 5 % CO_2_. For 3D culture of 3T3 cells, 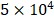 3T3 cells were mixed with 100 μL of 2 % (w/v) peptide solution in each well of 48-well plates. Then, 400 µL/well medium was gently added to trigger peptide gelation at 37 °C for 5 min. To optimize the pH (7.4) environment, the additional medium was changed three times within one hour. All samples were cultured in the humidified incubator at 37 °C with 5 % CO_2_.

#### Cell Viability

For cytotoxicity evaluation, 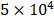 3T3 cells were seeded in peptide hydrogel with 400 µL of culture medium per 48-well plate. The viability of encapsulated 3T3 cells in the peptide hydrogel was examined by LIVE/DEAD Viability/Cytotoxicity Kit (Molecular Probe) and lactate dehydrogenase (LDH) assay. For LDH assay, at predetermined time intervals (1 and 3 days of incubation), 50 µL of cultured medium was mixed with 50 µL of CytoTox 96® Reagent in each well of a 96-well plate. After that, the microplate was incubated for 30 min covered with aluminum foil at room temperature. Finally, after the addition of 50 µL stop solution into each well, the absorbance wavelength at 490 nm was acquired by SpectraMax Plus 384 Microplate Reader. Furthermore, TritonX-100 was pretreated to each well and incubated for 90 min as a positive control group.

For negative control, the same number of 3T3 cells was cultured on a 48-well plate with 400 µL of culture medium without further treatment. The percentage of cell death is then calculated as:


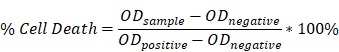


For LIVE/DEAD assay, the hydrogel was destroyed and incubated with 100 μL of staining solution containing 4 μM EthD-1 and 2 μM calcein AM for 45 min at 37 ^o^C. The staining solution was then aspirated and the cells were observed using fluorescence inverted microscope (Axiovert 40 CFL, Carl Zeiss).

#### Endothelial Tube-like Formation

To identify the angiogenic ability of released GFs *in vitro*, 80 µL of GF-depleted peptide hydrogel was introduced to a pre-cooled 96-well plate using chilled pipet tips. The gel was incubated for 30 min at 37 °C with 5 % CO_2_ to induce gelation. 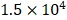 HUVECs were first seeded on GF-depleted peptide hydrogel with 100 μL of culture medium and incubated for 8 h. The experiments were designed into four groups including: (1) basal medium; (2) only HGF; (3) only VEGF; (4) both VEGF and HGF. The medium to which we treated were collected from the release buffer of peptide hydrogel with 200 ng VEGF or HGF incubated for 3 days at 37 °C. Finally, the differentiations of HUVECs were observed periodically for 8 h under an inverted phase contrast microscope. Angiogenesis Analyzer ImageJ (NIH) was employed for quantification and the images were taken in ten random fields per sample.

#### Entrapment of Defibrinated Blood

Microscopic morphology of the entrapment of defibrinated blood in the interwoven nanofibers was observed by scanning electron microscopy (SEM). Samples were prepared by fixation with 2.5 % glutaraldehyde in PBS at 4 °C for 4 h, and then serially dehydrated with ddH_2_O (twice), 30 % ethanol, 50 % ethanol, 70 % ethanol, 80 % ethanol, 90 % ethanol, 95 % ethanol and 100 % ethanol (twice). Samples in ethanol were then critically point dried using CO_2_ (Sorvall Critical Point Drying System). These dried hydrogel scaffolds were sputter coated with ∼8 nm Pt before examination using a field emission SEM (JEOL 6700F).

### *In vivo* Hemostatic Efficacy

The experimental protocol of *in vivo* study was approved by the Institutional Animal Care and Use Committee (IACUC) at Laboratory Animals Center in National Tsing Hua University. Male Sprague-Dawley rats purchased from BioLasco Taiwan Co., Ltd were used in the following surgical procedure. Each rat was placed on supine position and anesthetized with isoflurane in oxygen/air mixture with flow rate set at 2 L/min. The abdomen was then opened to expose the liver. On each of the two different sites in the same hepatic lobe, a 1 cm incision was created with a scalpel. For the experimental group, 2% QLK/LRK peptide solution was immediately injected through a 25-gauge needle. For the control group, the wound was treated immediately with 1X PBS. Videos were taken to qualitatively compare the hemostatic efficacy between the two groups.

### Incubation of Fertilized Eggs

Pathogen-free fertilized chicken eggs were obtained from Animal Health Research Institute (AHRI, Taiwan), and the *ex ovo* (shell-less) culture method was applied. On embryonic development day (EDD) 0, the eggs were incubated at 37.8 °C, 60 % humidity and turned automatically every 90 min. On EDD 3, egg shells were cracked in a sterile hood into square weigh boats covered with square petri dishes. Then, we dropped 200 units/mL penicillin/streptomycin on top of albumin to prevent infection. Fertilized embryos were then transferred to a water-jacketed CO_2_ incubator, set to 37.5 °C, 1 % CO_2_, and 100% relative humidity for further incubation. On EDD 6, ground egg shells were scattered around the embryos as a calcium source for bone maturation.

### *Ex ovo* Chick Chorioallantoic Membrane Assay

The experimental protocol of *ex ovo* study was approved by the Institutional Animal Care and Use Committee (IACUC) at Laboratory Animals Center in National Tsing Hua University. On EDD 8, GF-free hydrogel, 200 ng GF-loaded hydrogel, and 200 ng GF loaded on a filter paper 3 mm in diameter were applied on the outer surfaces of chorioallantoic membrane (CAM) at three different sites, respectively. Subsequently, we put back the embryos into the incubator and incubated for another 5 days to ensure the fully differentiation of CAM. Images of each groups and the surrounding CAM were captured by stereomicroscope (Zeiss Stemi 2000-C) immediately after applying the hydrogels, and recaptured on EDD 13. Subsequently, the proangiogenic response was assessed qualitatively by observing the vessels' morphological differences, and quantitatively by using NeuronJ, an online ImageJ plug-in. Next, the CAM tissue surrounding the hydrogels was collected and fixed with 4 % formaldehyde. The dehydrated CAM was then paraffin-embedded and sectioned. The sectioned tissue was stained with hematoxylin and eosin (H&E) for histopathological evaluation.

### Immunohistochemistry stain

Immunohistochemistry (IHC) staining was used to analyze the efficacy of angiogenesis in chick CAM. The CAM section was incubated at room temperature for 30 min, pre-treated with ice-cold ethanol for 10 min and then washed with PBS. The section was treated with blocking solution prepared by 4 % (v/v) FBS, 1 % (w/v) BSA, 0.25 % (v/v) Triton X-100, and 0.1 % (v/v) Tween 20 in 1X PBS at room temperature for 1 h, followed by incubation with primary antibody and secondary antibody at 4 °C overnight and at room temperature for 1 h, respectively. The counter staining was proceeded by DAPI at room temperature for 1 minute. Antibody dilution factors: primary anti-vWF antibody, 1:200; primary anti-αSMA antibody, 1:100; secondary goat anti-rabbit IgG H&L (Alexa Fluor 488) antibody, 1:200; secondary goat anti-rabbit IgG H&L (Alexa Fluor 594) antibody, 1:200; DAPI counter staining, 1:5000.

### Statistical Analysis

All results and data were expressed with mean ± standard deviation (S.D.) of the mean. Statistical significance was analyzed by Student *t*-tests to determine the significance of two individual data. A probability value of 95 % (p < 0.05, designated as * in figures), 99 % (p < 0.01, labelled as ** in figures) and 99.99 % (p < 0.001, designated as *** in figures) were used to compare the significance across each group.

## Results

### Physiochemical and Structural Characteristics of fSAP

**Figure 2:**
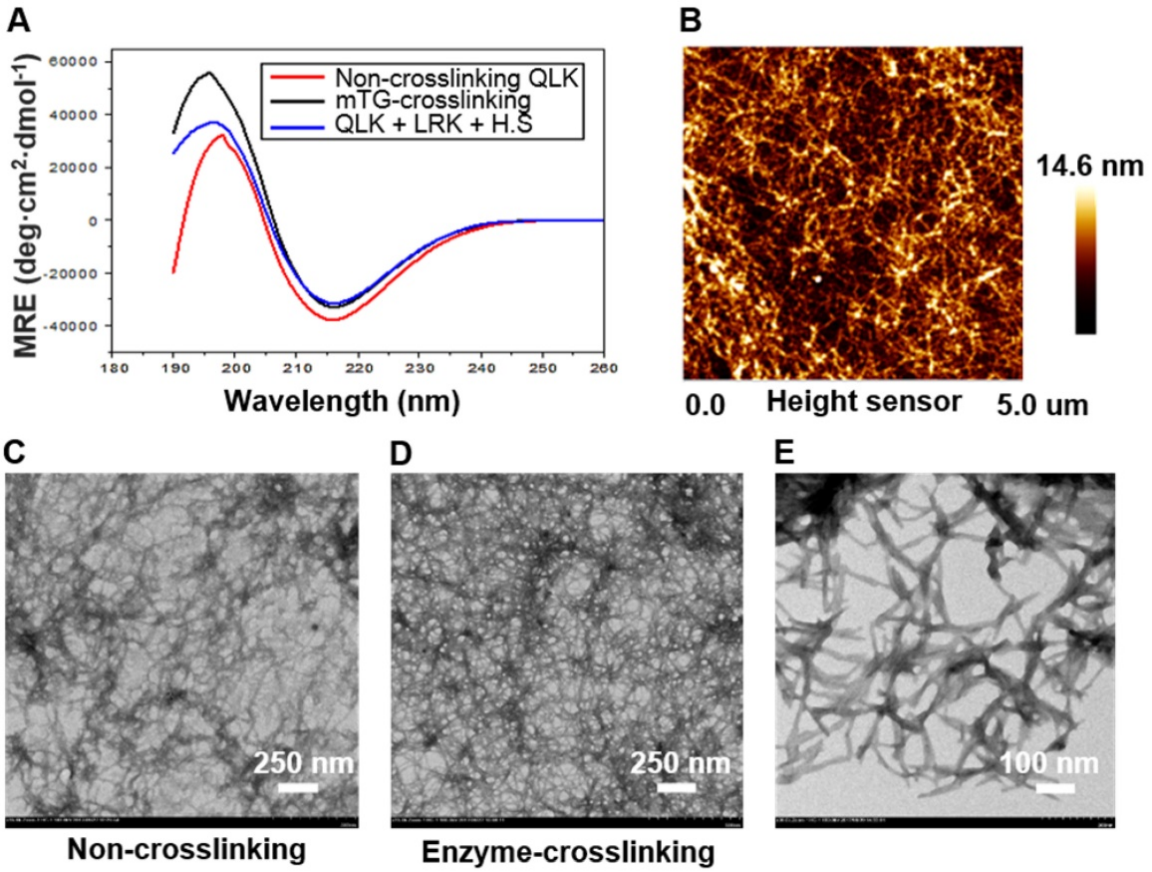
(A) The CD spectroscopy displayed the typical peaks, indicating the appearance of β-sheet structure in QLK sequences (red), QLK sequences after mTG crosslinking (black), and QLK sequences mixed with LRK sequences and HS (blue). (B) The representative AFM image of a three-dimensional fiber entanglement network of crosslinked QLK SAP. (C) The representative TEM images of 0.05 % (w/v) QLK SAP solution without mTG crosslinking. (D, E) 0.05 % (w/v) QLK SAP solution after mTG (20 U/mL) crosslinking with different magnifications.

According to the self-assembling mechanism of RADA-based peptide sequence, the β-sheet secondary structure played a critical role in the molecule assembling process 19. As a result, CD spectroscopy was used to demonstrate the appearance of β-sheet structure. Three experimental groups including QLK peptide, QLK peptide with enzymatic crosslinking, and QLK peptide mixed with LRK peptide and HS were examined to ensure the integrity of β-sheet secondary structure. The results indicated that a negative maximum peak at 216 nm and a positive maximum peak near 195 nm exhibit characteristics of β-sheet secondary structure (Figure 2A). TEM images were obtained to identify the morphological change of nanofiber microstructure before and after enzymatic crosslinking. Sparsely-wired fiber entanglements were observed before enzymatic crosslinking, indicating the stacking of β-sheet secondary structures self-assemble and organize into a mesh-like network (Figure 2C). After mTG crosslinking, more densely-compacted nanofibrous entanglements and more continuous self-assembling fibers were observed. Herein, the result demonstrated that the packing density of functionalized QLK peptide could be adjusted with the assistance of transglutaminase (Figure 2D). From the findings of TEM and AFM images, the interwoven nanofibers with diameters of 10-20 nm and pore sizes between 10-200 nm were similar to natural ECM fibers in both appearances and scales (Figure 2B-E).

### Investigation of Rheological Properties of fSAP Hydrogel

**Figure 3:**
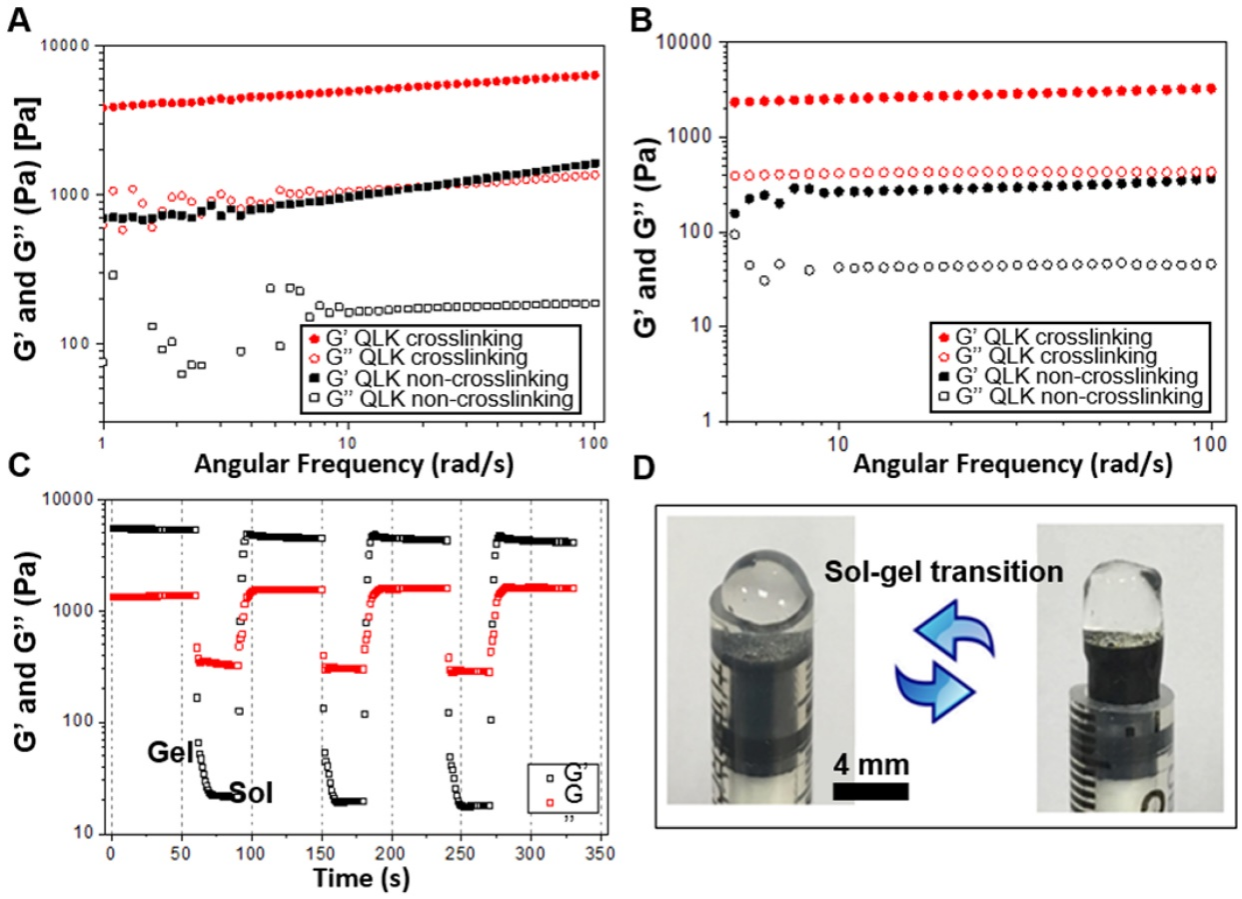
** Rheological properties of** peptide** hydrogel.** (A, B) Frequency-dependent (strain = 1 %, 1 Hz, 25 °C) oscillatory shear rheology of QLK self-assembling hydrogel and QLK/LRK self-assembling hydrogel (G' is the storage modulus representing the elasticity of a material and the ability of the material to store energy. G'' is the loss modulus related to the ability of the material to dissipate energy.) (C) The shear recovery property of QLK peptide hydrogel demonstrated by the continuous step strain (n = 6). (D) Representative gross photography showed the sol-gel transition process of QLK/LRK peptide hydrogel.

To test the mechanical stability and stiffness of the peptide hydrogel, frequency sweep and shear recovery measurements were conducted. Frequency-dependent oscillatory rheology of hydrogel measured at 25 °C showed a significant increase in mechanical stiffness after mTG enzyme crosslinking under investigative stimulation frequencies of shear rate. The storage modulus (G') of QLK hydrogel increased from 1000 Pa to 5000 Pa, while the G' of QLK/LRK hydrogel increased from 400 Pa to 2500 Pa (Figure 3A-B). At least a five-fold enhancement in rheological stiffness and the mechanical properties of QLK-functionalized hydrogel were adjustable with mTG treatment. The concentration of transglutaminase also had influence on the mechanical properties of the hydrogel. Basically, the storage modulus (G') of the hydrogel was increased with the increase of mTG concentration. It was measured at pressure levels of 1 kPa, 1.5 kPa, 3 kPa, 5 kPa with respect to the concentrations of 0 U/mL, 5 U/mL, 10 U/mL, 20 U/mL. Besides, the continuous step strain (1 % strain for 60 s → 50 % strain for 30 s → 1 % strain for 60 s) treatments demonstrated shear thinning and a potential shear recovery ability of QLK hydrogel (Figure 3C), suggesting the hydrogel was injectable and could be applied by syringes (Figure 3D).

### Characterization of Functionalized Peptide Hydrogel and the Interaction with GFs

**Figure 4:**
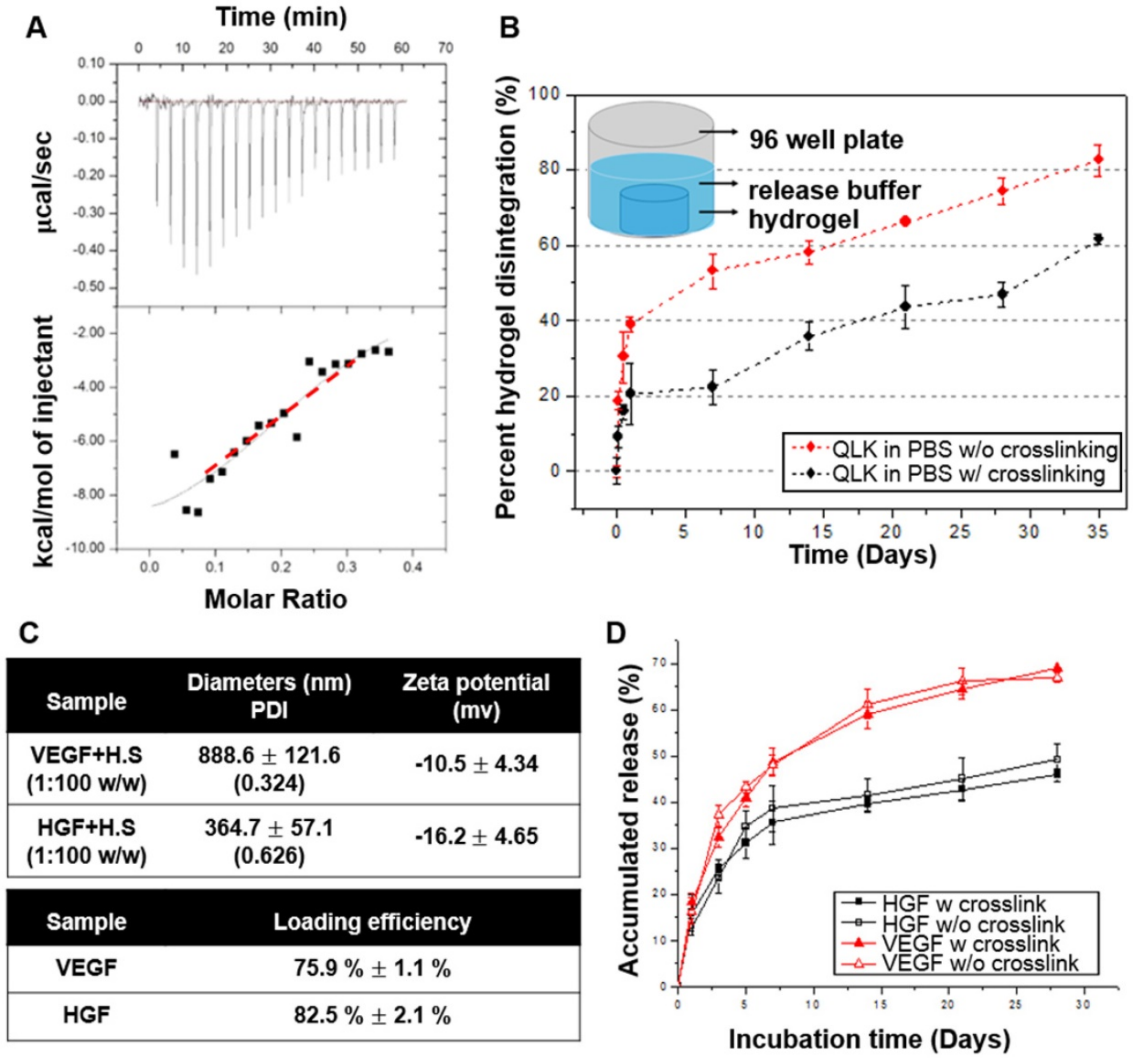
(A) Isothermal titration calorimetry evaluated the binding of HS to LRK peptide solution. The top graph indicated the heat released upon the addition of HS into LRK solution, and the bottom graph showed the integrated values (black squares) and the fitting line (red line) (binding constant Ka =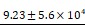 M^-1^). (B) Disintegration profile of 2 % (w/v) QLK hydrogel without mTG (red) and after mTG (20 U/mL) crosslinking (black), the crosslinking process delayed the dissociation rate of hydrogel scaffold. (C) The diameters, surface charges of the complexes of HS and VEGF, HGF respectively; the GFs loading efficiency were analyzed by ELISA kits. (D) *In vitro* cumulative release profiles of VEGF and HGF from GAG-assisted self-assembling QLK/LRK peptide hydrogel with or without mTG crosslinking. HGF (black) and VEGF (red) were released from peptide hydrogel scaffold in a sustained fashion for 28 days. Error bars indicated mean ± S.D. for total *n* = 6.

To test the binding affinity between HS and LRKKLGKA peptide sequence, the molecular interactions between LRK sequence and HS were studied by ITC. Heat changes were recorded when the increments of HS solution were titrated drop by drop into functionalized peptide LRK solution. The binding constant Ka =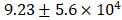 M^-1^ was then obtained by approximating the integrated data into a fitting curve (Figure 4A). The results verified that LRK peptide motif was a good HS binding consensus sequence.

To evaluate the stability of the peptide hydrogel in PBS buffer, Ninhydrin reagent solution was used to quantify the disintegration rate of peptide hydrogel *in vitro*. In the first three days of incubation, both groups showed initial degradation; only 20 % of the crosslinked hydrogel scaffold was disintegrated while the figure became 40 % in the non-crosslinked scaffold. After this initial period of rapid disintegration, the kinetics slowed down. Both groups presented behaviors of disintegration and resulted in a total disintegration of approximately 61% (crosslinked) and 82% (non-crosslinked) by 35 days (Figure 4B). The result revealed that the mTG crosslinking process not only reduced the disintegration of peptide hydrogel in the first few days but also maintained the *in vitro* stability of the hydrogel scaffold. The disintegration rate of hydrogel was slower in the crosslinking group when compared with that in the non-crosslinking group during the investigative time period. Next, we are interested in figuring out if different culture condition will result in different peptide disintegration rate. Thus, another group of peptides incubated in cell culture medium was also conducted for comparison. The disintegration curve showed that the peptide hydrogel degraded faster in the culture medium condition compared to that in PBS, especially in the group without crosslinking (Figure S1 in Supplementary Materials).

HS and heparin have been proved to have strong binding affinities with multiple GFs. Tekinay et al. showed that the binding constant between heparin with VEGF and heparin with HGF was 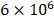 M^-1^ and 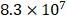 M^-1^, respectively 20. We mixed the VEGF/HGF and HS in a mass ratio of 1:100 to ensure a saturated binding of all the available GFs. DLS was used to measure the diameter and the zeta potential of GF/HS complex in 1:100 (w/w). The hydrodynamic diameters were 888.6 ± 121.6 nm (polydispersity index (PDI): 0.324) for VEGF and 364.7 ± 57.1 nm (PDI: 0.626) for HGF. From the data obtained from DLS, we found that the diameters of GF/HS complexes were larger than the pore size of the fibrous network evaluated in TEM (Figure 2E). The VEGF/HS complex had negative zeta potential of -10.5 ± 4.34 mV, and HGF/HS complex had more negatively of -16.2 ± 4.65 mV (Figure 4C).

It was the major challenge for scaffold-based GF delivery that how to maintain the release rate of incorporated GFs. ELISA kits for VEGF and HGF were used to quantify the amount of released VEGF and HGF in the supernatant. After the incorporation of GF/HS complex into peptide solutions and the formation into the hydrogels, the loading efficiency calculated was approximately 75.9 ± 1.1 % for VEGF and 82.5 ± 2.1 % for HGF, respectively. The *in vitro* release profiles of VEGF and HGF were depicted in Figure 4D, showing the time course of cumulative release of VEGF and HGF. The VEGF release profile demonstrated an initial release of 32 % on day 3. After 7 days, the release rate became nearly linear and the accumulated release was approximately 68 % by 28 days. On the other hand, the HGF release kinetics showed an initial release of 25% on day 3. After 5 days, the release rate also became significantly slowed down and the total release of approximately 46% by 28 days.

For comparison, the *in vitro* cumulative release profiles of VEGF and HGF from RADA16 peptide hydrogel without functional motifs modification was also documented (Figure S2 in Supplementary Materials). The result showed that GAG-assisted self-assembling QLK/LRK peptide hydrogel significantly extended the release time-span to about one month when compared to the non-GAG-assisted self-assembling RADA16 peptide hydrogel. These results demonstrated the ability of functionalized heparin binding sequence LRK to encapsulate VEGF/HS and HGF/HS complexes within QLK/LRK peptide hydrogel, while maintaining and delaying the release of GFs from the hydrogel scaffold.

### *In vitro* Regulation of Tube-like Formation by Endothelial Cells

**Figure 5:**
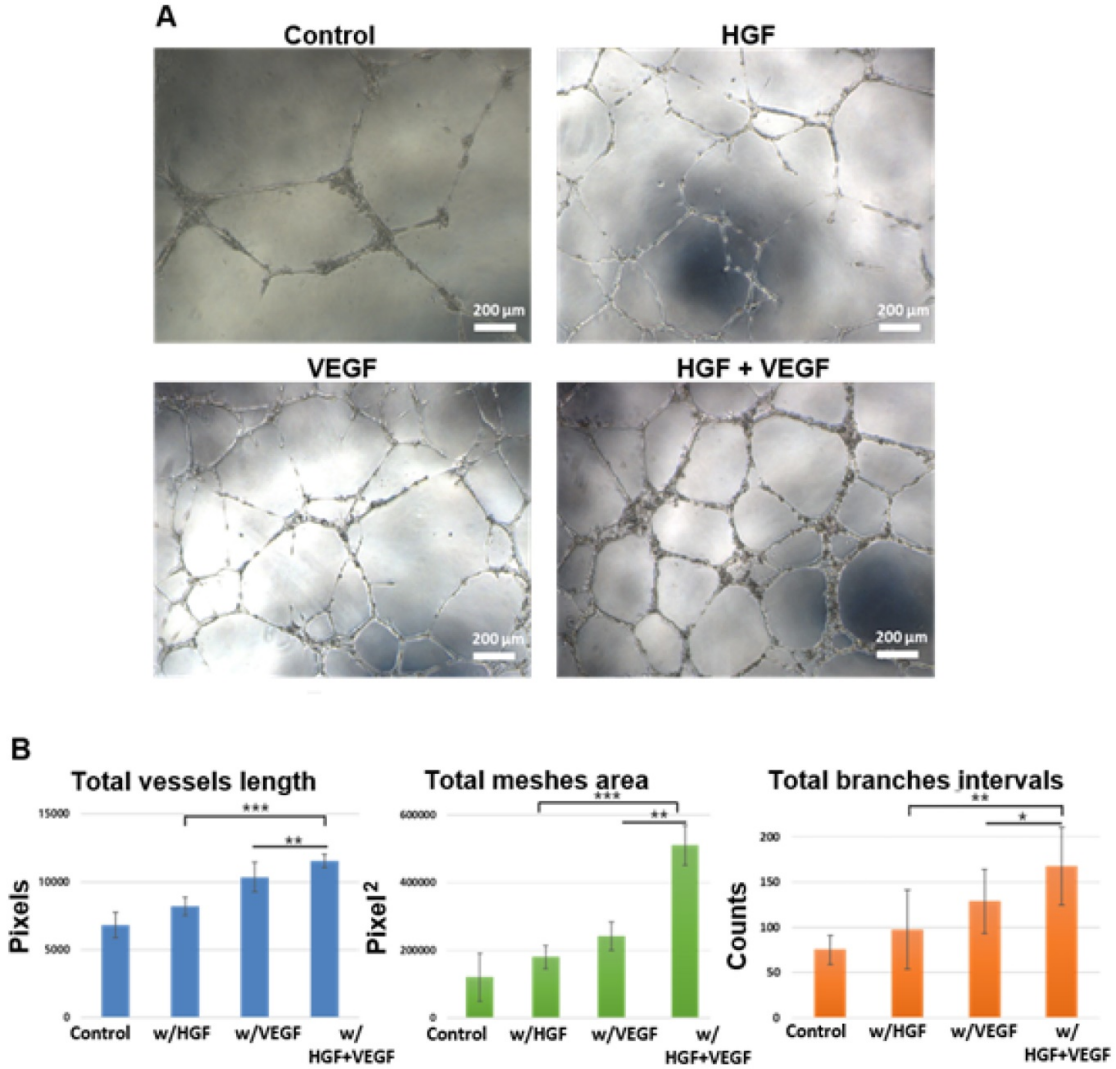
** The human umbilical vein endothelial cells (HUVECs) tube-like formation assay.** (A) The microscopic images of HUVECs tubular structure formation after 8 h of incubation in culture media under control, w/HGF, w/VEGF, and w/VEGF+HGF conditions. (B) The original images were analyzed by Angiogenesis Analyzer for ImageJ (NIH), and the quantified result of total vessels length, total meshes area, total branches intervals were calculated (all the values were normalized with the analyzed area). The results indicated VEGF and HGF synergistically enhanced the differentiation of HUVECs into tube-like structures. * indicates p < 0.05, ** indicates p < 0.01, and *** indicates p < 0.001. Error bars show ± S.D. for total n = 8.

The HUVEC tube formation assay was applied to verify the angiogenic ability of hydrogel-encapsulated GFs. Figure 5A shows the representative images of HUVECs cultured on peptide hydrogel in different medium conditions. Both VEGF and HGF groups showed a positive effect on tube-like formation of HUVECs compared to the basal medium control group. Notably, the supplementation of dual GFs, namely VEGF and HGF together, significantly promoted tube formation in these endothelial cells. Furthermore, the branching and interconnecting growth of these tubular structures were most obviously observed in the group with both GFs. Tube formation was quantified using Angiogenesis Analyzer for ImageJ (NIH). Total vessels length, total meshes area, and total branches intervals were obtained and normalized (Figure 5B). The *in vitro* cytocompatibility of peptide hydrogel scaffold was also performed. The results showed that the fSAP hydrogel was a highly biocompatible material without cell cytotoxicity (Figure S3 in Supplementary Materials).

### *In vivo* Hemostatic Efficacy

**Figure 6:**
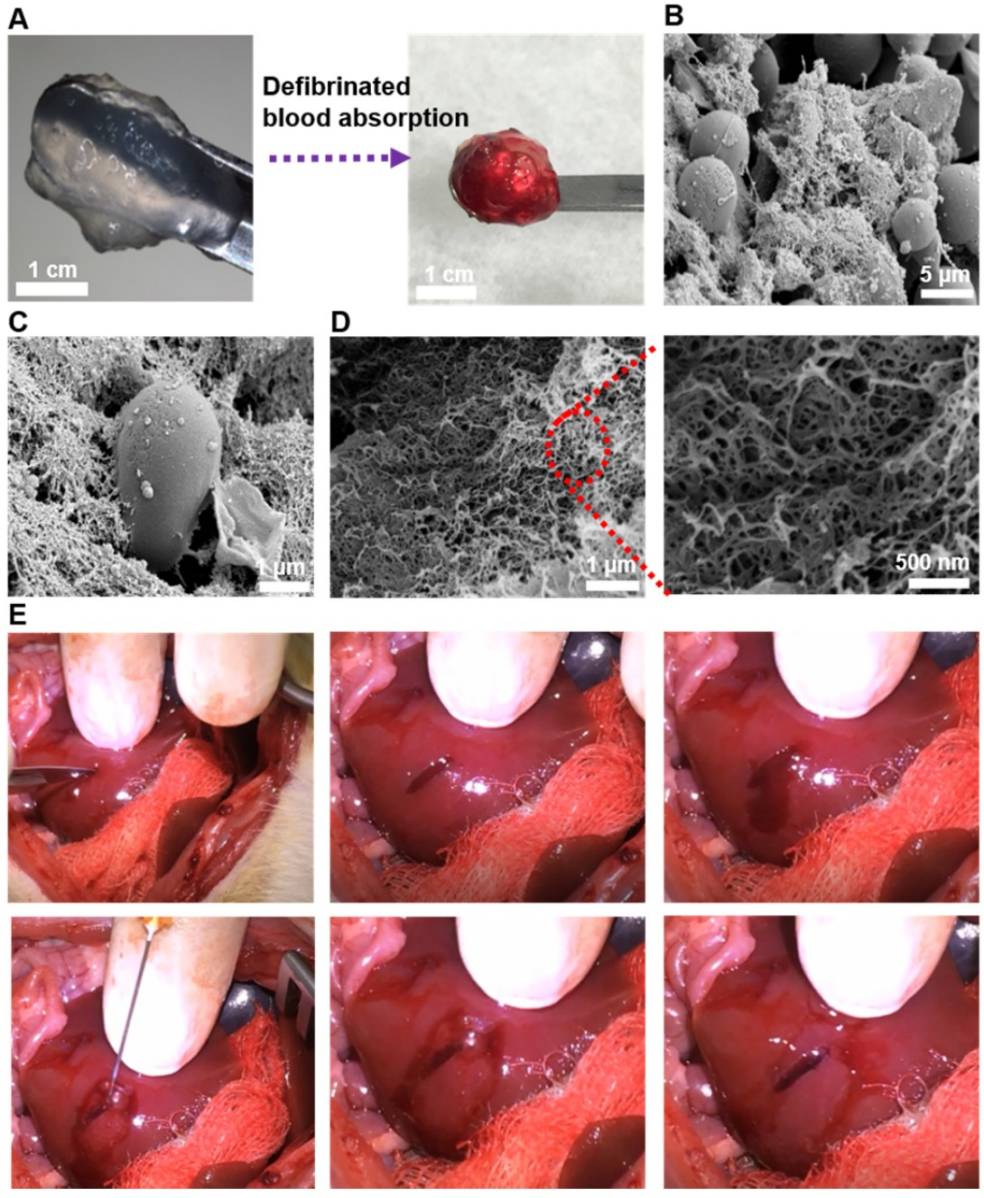
(A) Gross visualization of the fSAP hydrogel before and after the absorption of defibrinated blood. (B-C) The interaction between red blood cells and self-assembled fibers observed by SEM. (D) The three-dimensional and interpenetrating nanofibers could also be noticed in higher magnifications. (E) Pictures demonstrating hemostasis in rat liver after creation of a cutting wound. Rat liver was exposed and a sagittal cut was created by a scalpel to induce active bleeding. Then, 60 μL of 2 % (w/v) QLK/LRK peptide solution was injected by a 25G syringe into the wound, and hemostasis could be achieved within 10 seconds. After removing the excessive parts of hydrogel, an optically transparent self-assembling hydrogel with good conformation to the wound could be well observed.

SEM was used to observe the microstructure of SAP hydrogel after the absorption of blood. Defibrinated blood was selected to exclude the interference of fibrin. From its gross appearance, we could observe that large amount of blood was assimilated by the peptide hydrogel (Figure 6A). Furthermore, SEM images revealed physical entrapment of red blood cells in the interwoven network of nanofibers, suggesting a mimicry of natural clotting (Figure 6B-C). From the SEM pictures with higher magnification, three-dimensional and dense nanofiber-entanglements were also appreciated (Figure 6D). Subsequently, the efficacy of *in vivo* hemostasis was tested on rat liver. A 1-cm sagittal cut was created and treated with 2% QLK/LRK peptide solution. From gross observation in the video clip (unpublished data), hemostasis was achieved in less than 10 seconds and stabilized the local environment; moreover, rapid * in situ* gelation could also be noticed and the hydrogel was easily conformed to the lesion cavity (Figure 6E). On the other hand, it took more than 60 seconds to totally stop the bleeding in the PBS control group.

### Angiogenic Response in *ex ovo* Chicken CAM Assay

**Figure 7:**
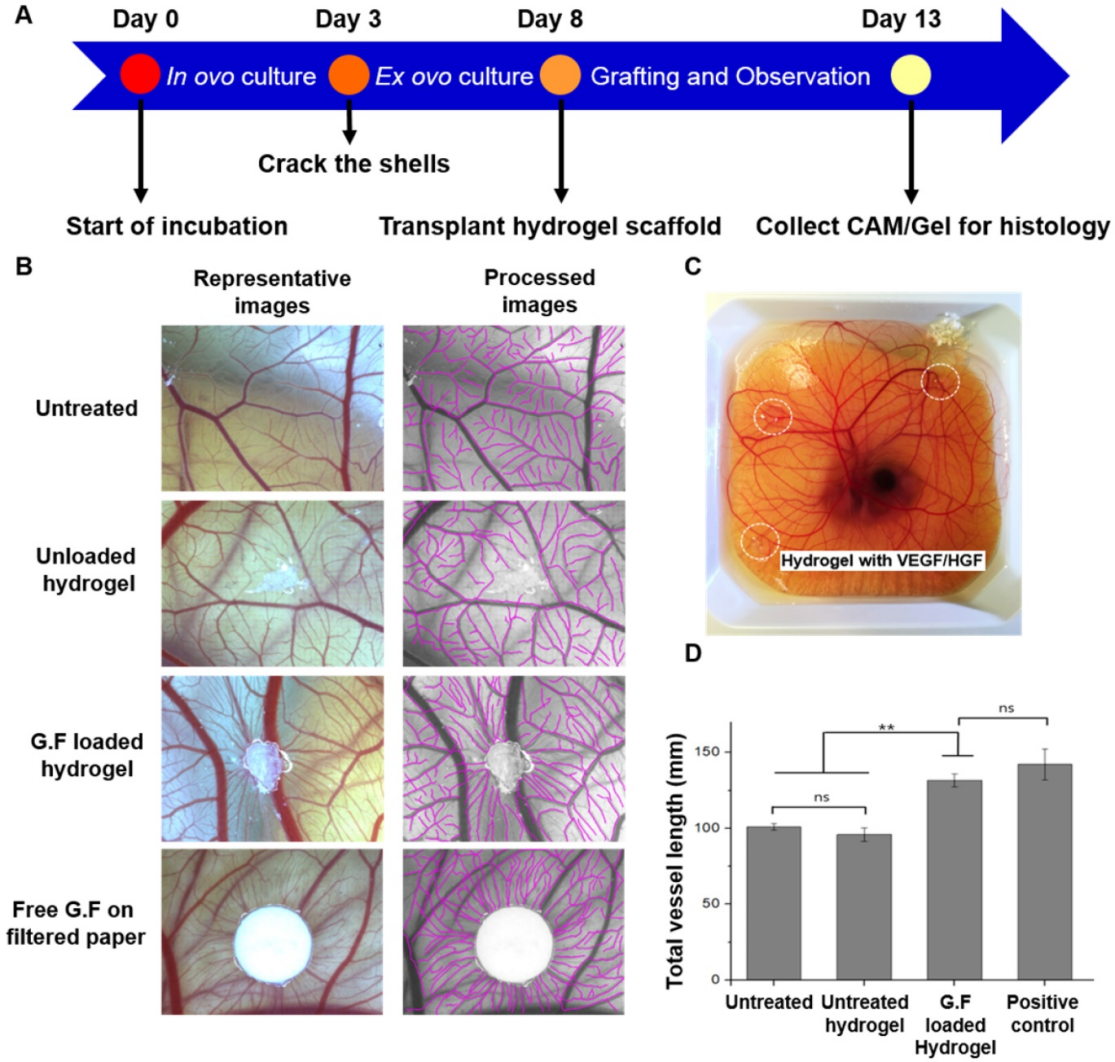
** Chick* ex ovo* culture using chicken CAM assay.** (A) Schematic time line of the *ex ovo* CAM procedure. (B) Representative images taken on EDD 13 showed CAM vascularization responsive to different treatments including untreated negative control, GF-free hydrogel, GF-loaded hydrogel, and free GF applied on filter paper as a positive control. The semi-automatic processed images were also provided for quantitative analysis. Scale bars indicate 2 mm. (C) Hydrogels loaded with 200 ng of VEGF and HGF, respectively, were grafted on the outer region of shell-less chicken embryo CAM. (D) Quantification of total vessel length surrounding the gel (the values were normalized with the analyzed area). * indicates p < 0.05, ** indicates p < 0.01, and NS denotes no significant difference. Error bars show ± S.D. for total n = 6.

**Figure 8:**
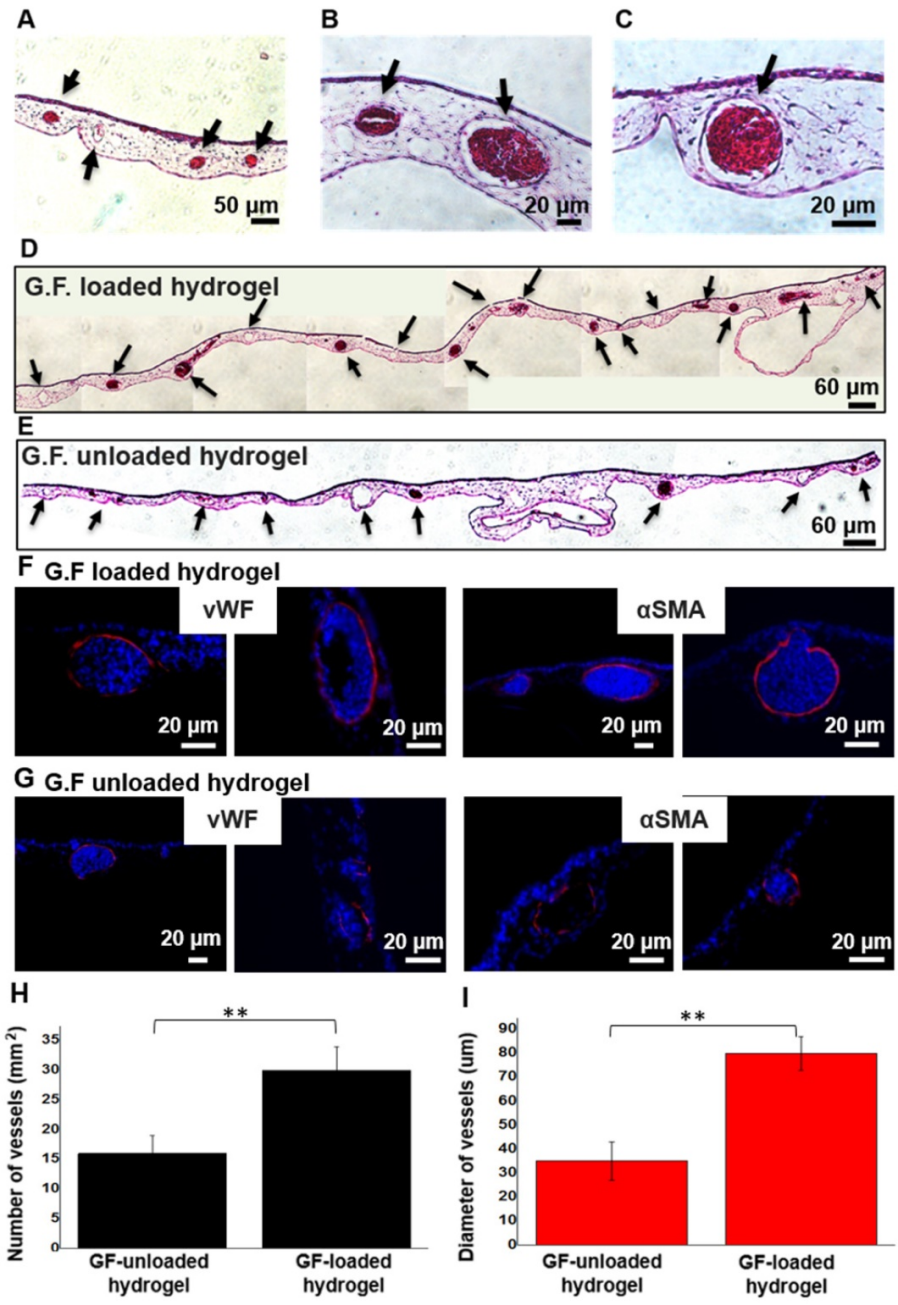
** Histological evaluation of CAM treated with GF-loaded and unloaded hydrogel using H&E and IHC staining.** (A-C) Capillaries, as indicated by black arrow, and red blood cells could be clearly seen in the magnified images of GF-loaded hydrogel group. (D) The stitched image of GF-loaded hydrogel treatment group. (E) The stitched image of pristine hydrogel treatment group. (F, G) Specific vWF and αSMA IHC staining of GF-loaded hydrogel and GF-unloaded hydrogel. (H, I) The quantitative analysis of vascular numbers and size of caliber vessels.

We chose chicken CAM, an established model for testing proangiogenic responses *in vivo*, to evaluate the angiogenic response on CAM vasculature after application of GF-loaded hydrogel. On EDD 13, the digital images qualitatively demonstrated that the hydrogel loaded with dual GFs and the treatment of free GFs significantly induced the surrounding vessels to grow toward hydrogel scaffolds in a spoke-wheel pattern when compared to pristine hydrogel group and untreated one (Figure 7B). Meanwhile, the results of quantitative analysis indicated the GF-loaded hydrogel induced greater total vessel lengths than (GF-) unloaded hydrogel and untreated counterparts (Figure 7D). It was noteworthy that the *in vivo* biocompatibility and safety could be evaluated at the same time, due to the high survival rate of chicken embryo with hydrogel grafting on CAM surface. In H&E staining, intact CAM vessels filled with red blood cells surrounding GF-loaded hydrogel could be seen clearly (Figure 8A-C). Furthermore, from the stitched images, larger caliber vessels and higher vascular numbers could be observed in the GF-loaded hydrogel group compared to that in the pristine hydrogel group (Figure 8D-E). In short, the CAM assay confirmed the proangiogenic property of designer SAP hydrogel immobilized with VEGF and HGF; furthermore, the result was in accordance with our previous findings in the HUVEC tube formation assay. We also checked the ultrastructure of newly developed vessels by IHC staining (Figure 8F-G). The results showed that the delivery of VEGF and HGF in the GF-loaded hydrogel could obviously promote angiogenesis when compared to GF-unloaded hydrogel, which was implicated by the presence of protein markers specific to endothelial cells and smooth muscle cells.

## Discussion

Hydrogels have been regarded as promising choices for tissue-engineering applications because they generally exhibit biomimetic matrix property, provide porous microenvironments with high water content for cell infiltration, and have a high permeability for oxygen, nutrients and other metabolites 21, 22. Biologically-derived natural polymers and synthetic-based natural ones are widely used for the creation of hydrogel scaffolds. Nevertheless, batch-to-batch variations and the risk of pathogen-induced immunogenic reactions hinder the use of biologically-derived polymers 23. On the other hand, synthetic-based natural polymers have more reproducible and adjustable physiochemical properties which make them more suitable to be fabricated into different hydrogel scaffolds. Among them, designer SAPs hold great promise owing to their inherent biocompatibility, biodegradability, and defined molecular arrangement. However, the fragile and unstable mechanical performance is the major limiting factor to utilize SAPs to act as regenerative constructs 24, 25. Inspired from the coagulation process, endogenous transglutaminase enzyme was selected to crosslink fSAP with the benefit of its resistance to proteolytic degradation and a relatively fast crosslinking reaction time 26, 27.

For self-complementary peptide sequences, the adoption of β-sheet arrangement is vital for further stacking and self-assembling into fibrous structure 28. From the results, both the supramolecular interactions and the β-sheet stackings are the main driving forces for the self-assembly of peptides. We have demonstrated that the formation of β-sheet structure was due to the alternating appearances of hydrophilic amino acids arginine (R) and aspartic acid (D), and the hydrophobic residue alanine (A); moreover, the β-sheet secondary structure was not interrupted after the conjugation of QLK and LRK functional motifs, enzyme-mediated process, and the addition of HS. QLK sequence self-assembled into fibrous network at pH = 7, and more condensed into continuous fibrous frameworks after mTG crosslinking. It was documented that for hydrogel matrices, the compactness of fiber microstructures determined its mechanical performance 10. Quantitative rheological measurements of QLK hydrogel showed a five-fold increase of storage modulus from 1000 Pa to 5000 Pa after mTG mediation. This magnitude could be adjusted to match variable degrees of suitable stiffness for a wide range of soft tissues.

We also found that after enzymatic crosslinking, the hierarchical three-dimensional supramolecular architectures closely mimic the real *in vivo* microenvironment of ECM fibrillary structures with porous construct in nanoscale 29. Furthermore, the hydrogel disintegration kinetics played a crucial role in determining whether the scaffold was suitable as the delivery vehicle for biomolecules or drugs 30. The results illustrated that the *in vitro* disintegration profile of crosslinked QLK hydrogel was significantly reduced. It seems like that the formation of covalent isopeptide bonds between free amine groups from lysine (K) and the γ-carboxamide groups from glutamine (Q) contributed to stabilize the QLK hydrogel and to enhance the mechanical property of the scaffolds. In the *in vivo* study on rat livers, peptide solution was directly injected into the bleeding site and immediately formed hydrogel * in situ*. We also showed that the optically transparent hydrogel easily conformed to lesion cavities and entrapped red blood cells and ECM proteins in place, reflecting the potential applications of SAP hydrogels in oozing wounds or even in minimally invasive surgical procedures. The therapy stopped bleeding without the use of cauterization, vasoconstriction, or chemical cross-linked adhesives. The self-assembling solution was nontoxic and non-immunogenic, and the breakdown products were mainly amino acids, which were tissue building blocks that could be used to repair the site of injury.

For the hemostatic mechanism of the fSAPs, QLK/LRK fSAP may rapidly self-assemble into flexible and entangled nanofibers. The newly formed nanofibers are responsible for hydrogelation after absorption to blood or body fluid. Factor XIII in blood is then converted by thrombin to an active transglutaminase protein Factor XIIIa with the presence of calcium ions during bleeding. Then, the activated factor XIII acts as fibrin stabilizer by the formation of intermolecular ε-(γ-glutamyl) lysine isopeptide bonding between acyl-donor lysine and acyl-receptor glutamine residues. This covalent crosslinking process is able to resist proteolytic degradation and is a relatively fast crosslinking reaction. Therefore, the peptide solution showed a more rapid and effective hemostatic agent via a combination of gelling blood and aggregated platelets. The above advantages make transglutaminase a potential candidate for enhancing self-assembling peptide hydrogel scaffold by rational crosslinking.

In chronic wounds, the angiogenic process is impaired due to that persistent inflammatory and excessive proteolytic conditions cause the cleavage of existing GFs and cytokines 31. Therefore, providing an ECM-mimicking milieu, which not only restores integrity of injured tissues but also serves as an appropriate reservoir for embedding therapeutic biomolecules, is an attractive approach to treat chronic ischemic diseases. However, conventional peptide building blocks such as RADA_16_ have no specific functional motifs for regulating cell fates and instructing host tissue regenerations. For therapeutic angiogenesis, delivering therapeutic proteins is the most straightforward strategy to induce proper vascularization. Among various GFs, VEGF is well-studied for its ability to stimulate neovascularization and to increase capillary permeability 32. Additionally, HGF is able to enhance epithelial regeneration and is now appreciated as an anti-inflammatory and anti-fibrotic molecule that could attenuate chronic disease progressions 33, 34. Moreover, both positively charged proangiogenic GFs and chemokines are discovered to have stable association with negatively charged HS, which have been demonstrated to create morphogen gradients to induce chemotaxis of endothelial cells 35, 36. In this study, the QLK/LRK hydrogel-based scaffold hold a network microstructure that not only provided a supporting scaffold for cells but also consisted of proteoglycans and bioactive proteins to guide cellular functions. By ITC measurement, we also revealed that peptides conjugated with designer heparin-binding consensus sequence LRK possessed a good binding affinity with HS. It indicated that the electrostatic attraction force and conformational considerations contributed to the good association affinity 37. Hence, the designer QLK/LRK hydrogel could sequester two positively charged angiogenic factors, VEGF and HGF, with high encapsulating efficiency. Notably, the HS containing-hydrogel scaffold slowly liberated VEGF and HGF in a sustained manner for 28 days *in vitro*. The delivery kinetics were mostly controlled by the charge of the complexes but not by the size of the complexes.

The slower release rate of HGF was in accordance with its higher binding affinity with heparin or HS than VEGF, and the more negative charge after complexation with HS. This prolonged and localized release of dual GFs was expected to overcome the major limitation of bolus GF injection approach, which often resulted in a rapid diffusion and proteolysis with low delivery efficiency 38, 39.

From previous studies, it was gradually becoming clear that the administration of single GF was not enough to achieve a desired therapeutic effect 40. We confirmed from the *in vitro* HUVEC tube-like formation assay that the combinational use of VEGF and HGF formed a more obvious capillary-like network than either GF used alone or the negative control group. The result pointed out that the bioactivity of VEGF and HGF still maintained after released from SAPs hydrogel. Additionally, the synergistic angiogenic effect of VEGF and HGF suggested that HGF might enhance endogenous VEGF expression and co-regulate their pathways during endothelial morphogenesis 41, 42.

The *ex ovo* CAM model offered a good accessibility to directly visualize vascular morphogenesis and evaluate the angiogenic potential of grafted materials. Meanwhile, several studies indicated that the results of vascular response on chicken CAM were comparable to the subcutaneous murine model; therefore, CAM model made a less sentient *in vivo* model to verify the potential for further translational research 43-45. After grafting hydrogel on CAM surface for 5 days, qualitative and quantitative analyses clearly demonstrated that proteoglycan-assisted delivery of VEGF and HGF could induce the converging growth of blood vessels toward fSAP hydrogel and increase the total vessel length around the implants. IHC staining results also supported this finding with strong expressions of vWF and αSMA. Taken together, we proved that the administration of elegantly designed SAP hydrogel could deliver embedded GFs in a sustained manner and promote angiogenesis. Therefore, it has the potential to serve as an effective hydrogel-based treatment for a broad range of soft tissue regeneration therapies, including surgical hemostasis, chronic ulcer, critical limb ischemia, ischemic stroke, and peripheral vascular disease, etc.

## Conclusion

We have successfully developed designer SAPs which 1) possessed the ability to form hydrogel * in situ*, 2) were crosslinked by endogenous enzymes, and 3) were able to achieve localized release of encapsulated GFs for therapeutic angiogenesis. RADA_16_ self-organized into β-sheet secondary structures under physiological environment and further composed the entangling fibers with the flanking of functional motifs: QQLK (QLK) and LRKKLGKA (LRK). Collectively, we presented that the hydrogel recapitulated the native structural and chemical features of ECM microarchitecture. The mechanical property and the stability of formed hydrogel could be enhanced after endogenous enzyme mediation, the hydrogel thus became a promising hemostatic agent. Furthermore, the adoption of proteoglycan HS served as a protecting carrier for sequestering dual GFs, *i.e.* VEGF and HGF, and maintained their release locally at injection site. The GF-loaded SAP hydrogel promoted vascular tube-like formation *in vitro* and angiogenesis *in vivo*. To conclude, the fSAP hydrogel not only paved the way toward an effective scaffold-based treatment for ischemic tissue diseases but also had a potential to incorporate other GAG-binding biomolecules such as interleukin-10, bone morphogenetic protein-2, and stromal-derived factor-1α for a broader range of tissue regeneration therapies**.**
