# Supplementary material for: Bioinspired Self-assembling Peptide Hydrogel with Proteoglycan-assisted Growth Factor Delivery for Therapeutic Angiogenesis
Source: Theranostics. 2019 Sep 21;9(23):7072–87. doi: 10.7150/thno.35803 (PMC6815956; doi:10.7150/thno.35803)
Supplement: Supplementary file 1 — Supplementary figures and tables. [file thnov09p7072s1.pdf]

Bioinspired Self-assembling Peptide Hydrogel with Proteoglycan-assisted Growth Factor Delivery for Therapeutic Angiogenesis

Lu-Chieh Huang[1], *Huan-Chih Wang*[2,3], *Liang-Hsin Chen*[1], *Chia-Yu Ho*[1], *Pei-Hsuan Hsieh*[1], *Ming-Yuan Huang*[4,5], *Hsi-Chin Wu*[6], *Tzu-Wei Wang*[1*]

[1] Department of Materials Science and Engineering, National Tsing Hua University, Hsinchu, Taiwan

[2] Division of Neurosurgery, Department of Surgery, National Taiwan University Hospital, Taipei, Taiwan

[3] College of Biological Science and Technology, National Chiao Tung University, Hsinchu, Taiwan

[4] Department of Medicine, Mackay Medical College, New Taipei City, Taiwan

[5] Department of Emergency, Mackay Memorial Hospital, Taipei, Taiwan

[6] Department of Materials Engineering, Tatung University, Taipei, Taiwan

[*]Corresponding Author:

Tzu-Wei Wang, Ph.D.

Professor

Department of Materials Science and Engineering

National Tsing Hua University

101, Section 2, Kuang-Fu Road, Hsinchu, Taiwan 30013, R.O.C.

Tel: +886-3-5715131 ext. 33856 Email address: twwang@mx.nthu.edu.tw

Supplementary Materials

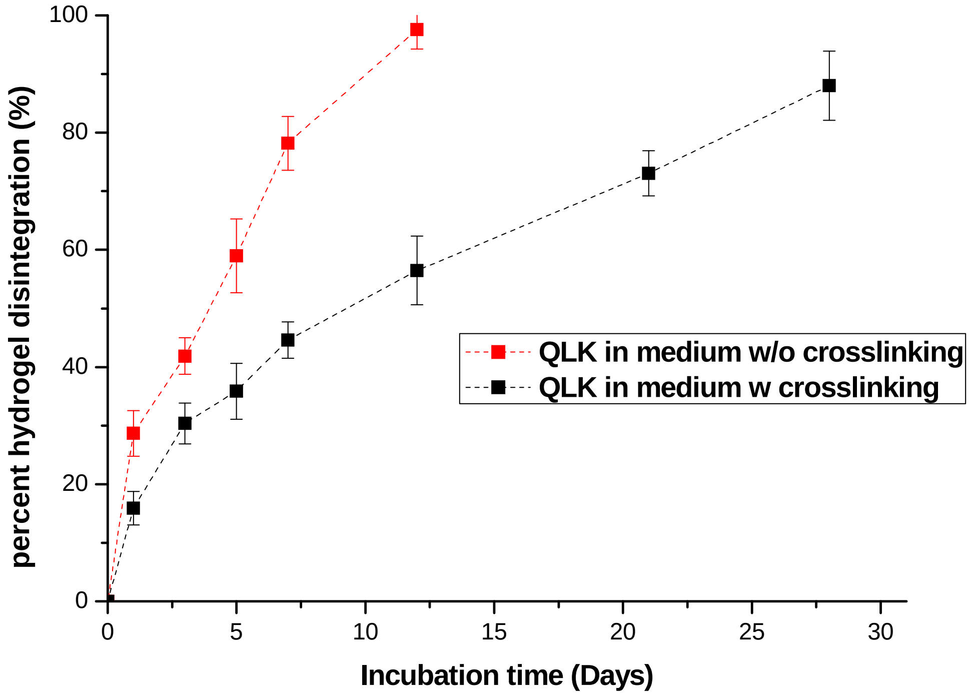

Figure S1. Disintegration profile of 2 % (w/v) QLK hydrogel in the cell culture medium without mTG (red) and after mTG (20 U/mL) crosslinking (black). Error bars show ± S.D. for total n = 6

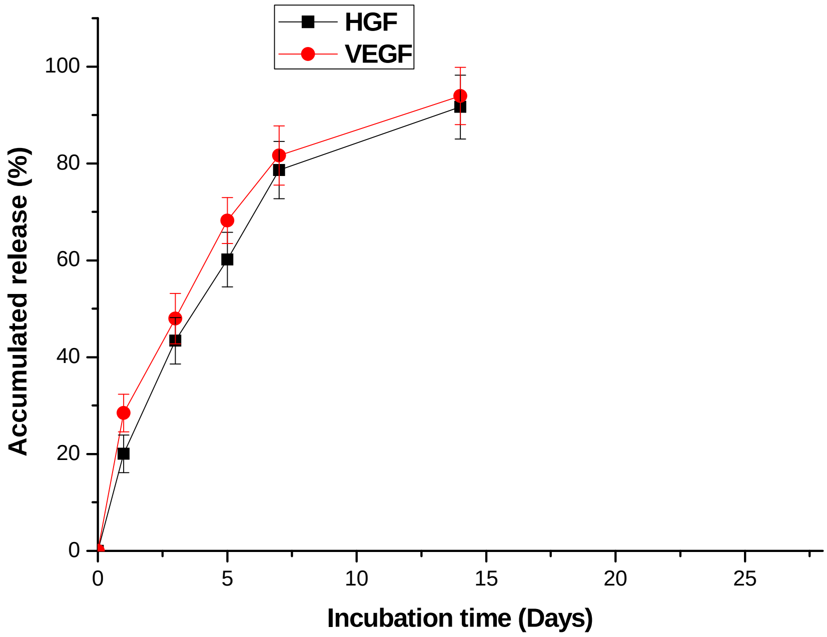

Figure S2. *In vitro* cumulative release profile of VEGF and HGF from RADA16 peptide hydrogel without functional motifs modification. The result suggests that GAG-assisted self-assembling QLK/LRK peptide hydrogel can significantly extend the release time-span to about one month as demonstrated in Figure 3D when compared to the non GAG-assisted self-assembling RADA16 peptide hydrogel. Error bars show ± S.D. for total n = 6

In vitro cytocompatibility of peptide hydrogel scaffold

To provide a proper environment that supports encapsulated cells to migrate, proliferate, and differentiate within the peptide hydrogel, the biocompatibility of QLK/LRK SAP hydrogel is a critical issue. To test the encapsulated 3T3 cells viability and cytotoxicity of hydrogel scaffold, Live/Dead and LDH assays were performed. According to the results of Live/Dead staining, living cells were stained with cell-permeant calcein AM, which showed green fluorescence. On the other hand, dead cells were stained with red fluorescent EthD-1. As such, the results showed that living 3T3 cells were dominant along with only few red fluorescence-stained dead cells after 3 days of cultivation in three-dimensional peptide hydrogel (Figure S1A-C). Quantitative analysis of cell death percentage in QLK/LRK hydrogel after 1 and 3 days of incubation was as well conducted by LDH assay. The LDH enzyme released into the media from disrupted cells was used as a biomarker for cellular cytotoxicity. The percentage of dead cells cultured within hydrogel stayed low compared to that incubated on tissue-culture polystyrene after 1 and 3 days cultivation (Figure S1D). In brief, the results demonstrated a good biocompatibility and low material-cytotoxicity of QLK/LRK peptide hydrogel.

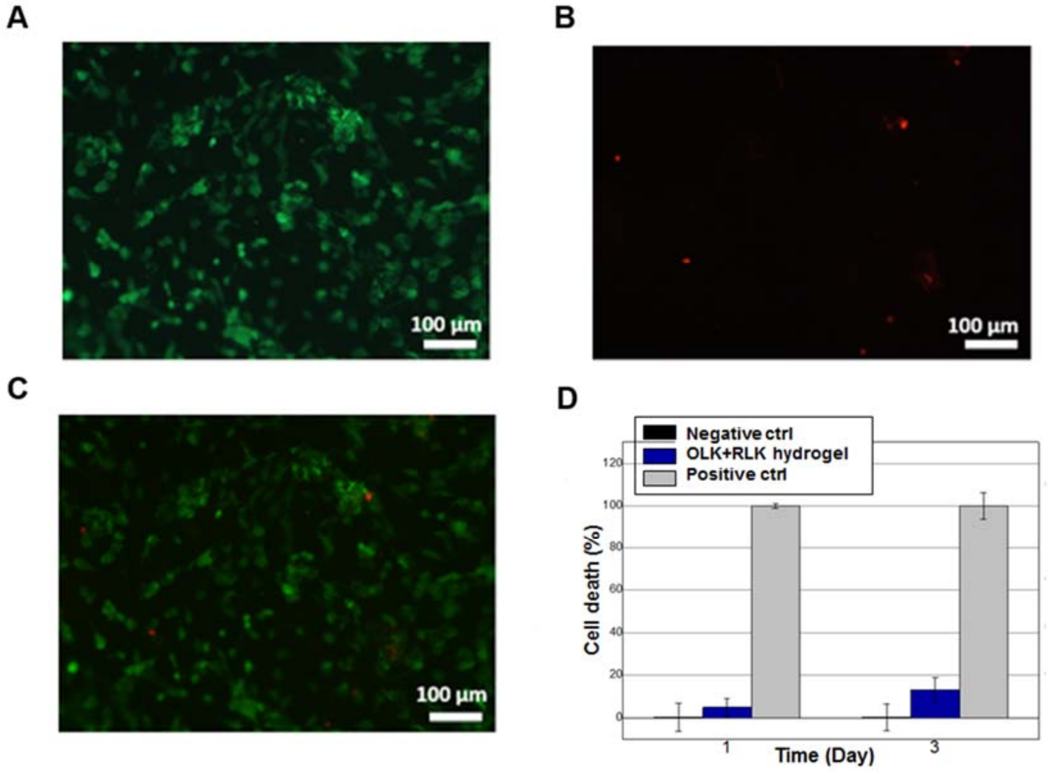

Figure S3. Cell viability and cytotoxicity of 3T3 fibroblasts embedded in three-dimensional self-assembling hydrogel of QLK/LRK peptide were assessed by Live/Dead staining at day 3 after incubation (A-C) and by LDH assay after cultured for 1 and 3 days (D). The results revealed a good biocompatibility and low material-cytotoxicity of QLK/LRK peptide hydrogel scaffold. Error bars show ± S.D. for total n = 6.
